# Functional and anatomical outcomes after primary lens-sparing pars plana vitrectomy for Stage 4 retinopathy of prematurity

**DOI:** 10.4103/0301-4738.53050

**Published:** 2009

**Authors:** Pramod Bhende, Lingam Gopal, Tarun Sharma, Aditya Verma, Rupak Kanti Biswas

**Affiliations:** Sri Bhagwan Mahaveer Vitreoretinal Services, Medical and Vision Research Foundations, Sankara Nethralaya, Chennai, India

**Keywords:** Lens-sparing vitrectomy, retinopathy of prematurity, tractional retinal detachment

## Abstract

**Background:**

While lens-sacrificing vitrectomy is the standard approach to manage Stage 5 retinopathy of prematurity (ROP), scleral buckling has been used to manage some cases of Stage 4. Lens-sparing vitrectomy was popularized by Maguire and Trese in selected cases of Stage 4 disease.

**Purpose:**

To assess the functional and visual outcomes after primary lens-sparing pars plana vitrectomy for Stage 4 ROP.

**Materials and Methods:**

In a retrospective, interventional, consecutive case series, the records of 39 eyes of 31 patients presenting with Stage 4 retinal detachment secondary to ROP who underwent primary two or three-port lens-sparing vitrectomy from January 2000 to October 2006 were evaluated. The outcomes studied at the final follow-up visit were the retinal status, lens and medial clarity and visual acuity. Favorable anatomical outcome was defined as the retinal reattachment of the posterior pole at two months after the surgery; and favorable functional outcome was defined as a central, steady and maintained fixation, with the child following light.

**Results:**

At mean follow-up of 15 months, 74% of the eyes had a favorable anatomical outcome with single procedure. The visual status was favorable in 63%. The lens remained clear in all the eyes at the last follow-up, and the media clarity was maintained in 87%. Intraoperative complications included vitreous hemorrhage, pre-retinal hemorrhage and retinal break formation.

**Conclusions:**

Lens-sparing vitrectomy helps to achieve a favorable anatomical and functional outcome in selected cases of Stage 4 ROP.

Anatomical and visual outcomes in eyes undergoing surgery for Stage 5 retinopathy of prematurity (ROP) are generally poor.[1] Surgical interventions for retinal detachment associated with advanced ROP in the form of scleral buckling,[2] or vitrectomy usually fail to obtain foveal formation, resulting in subnormal vision. Although the optimal time for surgical intervention is often difficult to determine, patients with Stage 4a ROP may have their retinas reattached prior to clinically detectable macular detachment, thus allowing for better visual rehabilitation and possibly reduce the risk of postoperative macular distortion and heterotopia.[3–5] Investigators utilizing two- or three-port lens-sparing vitrectomy (LSV) have reported good anatomical and functional success rates with adequate follow-up in several large series.[5–8] In the current study, we report our results of primary LSV for Stages 4a and 4b ROP[9] performed at a tertiary eye care center in India.

## Materials and Methods

Institutional review board approval was duly obtained to review the patient data before the start of the study. A detailed prenatal and postnatal and treatment history was taken. All the infants had a detailed systemic evaluation by a neonatologist. The eyes were dilated with combination of 0.75% tropicamide and 2.5% phenylephrine as two instillations at 15 min-interval. The detailed ocular examination included the anterior segment evaluation (including the lens clarity); the status of vitreous, the optic disc, and the retina, (with special reference to the macular status).

All the surgeries were performed by two surgeons (LG/PB). General anesthesia was administered by trained pediatric anesthetists. Special precautions were taken to keep the child warm. After anesthesia, a thorough fundus examination was performed using indirect ophthalmoscope with scleral depression to assess the extent of retinal detachment and for the selection of location of sclerotomy.

Where two-port vitrectomy was decided upon, infusion and illumination were provided with the infusion light pipe (Catalog no. 56.14, Synergetics Inc. MO USA). For the three-port vitrectomy procedure, routine sclerotomies were made for infusion, light pipe and the active instrument. Sclerotomies were made 0.75-1.25 mm from limbus. The clock meridian of the sclerotomies was selected based on the configuration of the retinal detachment. In cases with tractional fold in the temporal periphery (anterior Zone 2), all the sclerotomies were made in the nasal half of the sclera, with the surgeon sitting on the side opposite to the eye being operated.

While making sclerotomies for LSV, unlike conventional adult vitreoretinal (VR) surgeries, the microvitreoretinal (MVR) blade was directed straight vertically downward and parallel to the visual axis. MVR blade was also used to cut and to create an opening in trans-vitreal membranes in the meridian of sclerotomy for smooth and traction-free entry of blunt instruments such as vitreous cutter and endoilluminator. During the surgery the instruments were kept as vertical as possible and precaution was taken not to cross the midline, so as to avoid the lens touch. It was necessary to swap the instruments between two superior sclerotomies to complete the surgery.

All the surgeries were performed either using *Accurus surgical system* (Alcon Inc. Fort Worth TX USA) or *Millennium microsurgical system* (Bausch and Lomb Inc. Rochester NY USA). Visualization was accomplished with binocular indirect ophthalmo microscope (BIOM system, Oculus, Wetzlar, Germany) or pediatric Lander lens system.

The posterior hyaloid face was often attached and an attempt was made to separate it over the disc and retina to the extent possible. As much relief of the pre-retinal traction as possible was achieved. Elevated bleeders were diathermised. The extent of the surgery varied widely depending upon the configuration of the detachment and the extent and location of fibrous proliferation. The sclerotomies were closed using 7-0 vicryl sutures. At conclusion of the surgery, fluid-air exchange was not performed as a routine procedure. Postoperatively topical steroid and cycloplegic drops were used for a period of six weeks.

Postoperatively the operated eye was evaluated with a binocular indirect ophthalmoscope for medial clarity, status of retina and any recurrent fibrosis. Ultrasonography was done periodically, to find out the retinal status if vitreous hemorrhage precluded fundus visualization. Repeat surgical intervention was done if required after three to four weeks.

Anatomical success was defined as total reattachment of the retina or at least posterior polar reattachment. Visual status was evaluated based on the age at assessment-fixing and following behavior; with Leas symbols; or with Snellen's visual acuity chart. Intraocular pressure was measured with tono-pen, whenever possible.

## Results

The demographics and surgical outcome of the study group are listed in Tables 1 and 2. Thirty-nine eyes of 31 patients that underwent primary LSV for Stage 4a or 4b ROP formed the subjects of this study. There were 20 male children (25 eyes) and 11 female children (14 eyes). Bilateral LSV was carried out in eight patients (26%). The mean birth weight was 1242 g (range 650–2250 g) and the mean gestational age at birth was 29 weeks (range 24 – 34 weeks). The mean age at presentation to us was 37.6 weeks (range 32–52 weeks) and the mean age at the time of surgery was 42 weeks (range 36–57 weeks). Thirteen of the 31 babies were born after caesarian section while the rest were born after normal vaginal delivery.

**Table 1:** General patient data: 39 eyes of 31 patients

Eye	Sex	BW	GA	PCA	AP	AS	MC	Eye	ROP	VH	PH	TRD	IOC	RS2	RSL	Vn
1	F	1000	28	39	39	39	2,6	OS	4b	N	N	7	0	3	3	2
2	M	1200	27	37	33	37	6	OS	4a	N	N	4	1	1	2	1
3	M	900	26	41	36	41	3,6	OD	4a	N	Y	1	1	2	1	1
4	M	1070	29	38	33	38	1,3,6	OS	4a	N	N	2	0	1	1	2
5	F	2040	34	48	46	48	3	OD	4b	N	N	7	0	3	3	2
6	M	1510	31	42	37	42	3,6	OS	4b	N	N	7	1	2	2	1
7	M	1320	30	44	37	44	1,3,6	OD	4b	N	N	6	1	1	1	1
8	M	1080	28	45	36	45	1,3,6	OS	4a	N	N	4	1	4	5	2
9	M	1080	28	47	36	47	1,3,6	OD	4a	N	N	4	2	4	5	2
10	M	2250	32	41	39	41	2,3,6	OD	4b	N	Y	7	2	4	6	2
11	F	1100	28	40	33	40	2,6	OS	4a	N	N	3	0	1	1	1
12	M	750	25	42	42	42	1,3,6	OD	4a	N	N	1	0	1	1	1
13	M	750	25	43	42	43	1,3,6	OS	4a	N	N	1	0	1	1	1
14	M	1075	28	43	39	43	1,2,3	OS	4b	N	N	7	1	3	3	2
15	M	1110	28	42	34	42	1,3,6	OS	4a	N	Y	2	1	2	1	1
16	F	1600	30	46	44	46	3	OD	4a	N	N	4	0	2	1	1
17	F	960	28	36	33	36	2,3	OS	4a	Y	N	3	1	1	1	1
18	M	1040	28	44	43	44	2,3	OS	4a	N	N	1	0	LTF	LTF	
19	M	950	27	57	52	57	3,6	OD	4a	N	N	1	0	2	2	1
20	F	1270	30	43	36	43	2,3	OD	4a	N	Y	3	0	2	1	2
21	F	1350	30	42	38	42	1,3	OD	4a	Y	Y	5	1	1	1	1
22	F	1350	30	44	38	44	1,3	OS	4a	Y	Y	5	0	1	1	1
23	M	1500	32	41	37	41	1,2,3	OD	4b	N	N	7	3	3	3	2
24	M	1500	32	42	37	42	1,2,3	OS	4b	N	N	7	3	1	1	1
25	M	1550	30	44	34	44	1,2	OS	4b	N	N	6	0	2	1	1
26	M	1500	34	41	38	41	2,3	OD	4a	N	N	2	1	5	6	2
27	M	1232	31	42	40	42	2,3	OD	4a	N	N	4	0	2	2	1
28	M	1232	31	43	40	43	2,3	OS	4a	N	Y	4	0	2	2	1
29	M	1335	26	41	40	41	3,6	OD	4b	N	N	1,7	0	2	1	2
30	F	650	24	38	36	38	1,3,6	OS	4a	N	N	2	0	1	1	1
31	F	650	24	39	36	39	1,3,6	OD	4a	N	N	2	0	1	1	2
32	F	1000	27	40	32	40	2,3,6	OS	4a	N	N	3	3	1	1	1
33	F	1200	30	42	34	42	1,2,3,6	OS	4a	N	N	5	1	1	1	1
34	F	1200	30	45	34	45	1,2,3,6	OD	4a	N	N	5	0	1	1	1
35	M	1350	30	39	38	39	1,2,3,6	OD	4a	N	N	3	0	6	1	1
36	M	1350	30	40	38	40	1,2,3,6	OS	4a	N	Y	3	0	2	2	1
37	M	1500	30	44	34	44	2,3	OS	4a	N	N	5	4	3	4	2
38	M	1970	32	43	38	43	2,3,6	OD	4a	N	N	1	0	1	1	1
39	F	970	26	42	41	42	1,2,3,6	OS	4a	N	N	1	0	3,6	5	2

No = Serial number (number of patient); Eye = Number of eye; Sex = F (Female), M (Male); BW = Birth weight in grams; GA = Gestational age in weeks; AP = Age at presentation in weeks (post-conceptual); AS = Age at surgery in weeks (post-conceptual); MC = Medical condition at birth (from NICU records) 1 = Septicemia, 2 = Hyperbilirubinemia, 3 = Respiratory distress syndrome, 4 = Hyperglycemia, 5 = Hypoglycemia, 6 = Others; Eye = OD (Right eye), OS (Left eye); ROP = Stage of retinopathy of prematurity at surgery (4a or 4b); PH = Preretinal hemorrhage preoperatively (Y = Present, N = Absent); VH = Vitreous hemorrhage preoperatively (Y = Present, N = Absent); TRD = Location of fractional retinal detachment (1 = Temporal, 2 = Nasal, 3 = Superior, 4 = Inferior, 5 = Peri-papillary, 6 = Macular drag, 7 = Posterior pole); LTF = Lost to follow-up; IOC: Intraoperative complications (0 = No complications, 1 = Minimal bleeding, 2 = Moderate to severe bleeding hampering completion of surgery, 3 = Retinal break, 4 = Corneal clouding). RS2 = Retinal status at two months follow-up (1 = Attached totally, 2 = Attached posterior pole, 3 = Detached posterior pole, 4 = Total RD, 5 = Closed funnel RD, 6 = Diffuse Vitreous hemorrhage); RSL = Retinal status at last follow-up visit (1 = Attached totally, 2 = Attached posterior pole, 3 = Detached posterior pole, 4 = Total RD, 5 = Closed funnel RD, 6 = Diffuse Vitreous hemorrhage); Vn = Vision at the last follow-up visit (1 = Central, Steady, Maintained; 2 = Not Following Light 3 = Not assessed)

**Table 2:** Demographic data and surgical outcome

Profile	Total	Stage 4a	Stage 4b
Number of eyes/Number of patients	39/31	29/23	10/8
Gender			
Males	25 eyes	17 eyes	8 eyes
Females	14 eyes	12 eyes	2 eyes
Mean gestatlonal age (weeks)/range	29 wks/24-34 wks	28.5 wks/24-34 wks	30.3 wks/26-34 wks
Mean birth weight (grams)/range	1242 g/650-2250g	1150.5 g/650-1970g	1 508 g/1 000-2250 g
Age at presentation (weeks)/range	37.6 wks/32-52 wks	35.7 wks/32-52 wks	38.5 wks/34-46 wks
Age at surgery (weeks)/range	42 wks/36-57 wks	42.2 wks/36-57 wks	42.5 wks/39-48 wks
Follow-up (months)/range	15 mth/2-55mth	13.6 mth/2-55mth	18.6 mth/5-48mth
Preoperative status			
Rubeosis (%)	6 (15)	6 (20)	0
Vitreous hemorrhage + Preretinal hemorrhage (%)	2 (5)	2 (7)	0
Relatively florid neovascularization (%)	9 (23)	7 (24)	2
Preoperative retinal ablation (%)	34 (87)	26 (90)	7
Adequate relief of traction (%)	37 (95)	27 (93)	9
latrogenic breaks (%)	3 (8)	1 (3)	2
Postoperative status	(n = 38)	(n = 28)	(n = 10)
Anatomic success (one procedure) (%)	28 (74)	23 (82)	5
Anatomic success (final) (%)	34 (89)	27 (96)	7
Favorable vision (one procedure) (%)	24 (63)	20 (71)	4
Favorable vision (final) (%)	30 (79)	24 (86)	6

Thirty-four eyes (87%) had undergone peripheral retinal ablation in the form of laser photocoagulation or cryopexy while five eyes presented to us with Stage 4a or b but without any prior treatment.

*The status of fellow eye*: Eight eyes underwent bilateral LSV. Fourteen out of the remaining 23 eyes had regressed ROP following laser photocoagulation; six eyes with Stage 4 (a or b) were not operated due to various reasons, while one eye had undergone scleral buckling elsewhere. Two eyes were in Stage 5 ROP.

Anterior segment examination revealed rubeosis in six eyes (15%) preoperatively, of which three eyes had pupils resistant to dilatation.

Preoperatively, intraocular hemorrhage was noted in 13 eyes (33%), preretinal hemorrhage in eight eyes; vitreous hemorrhage in three eyes and combined preretinal and vitreous hemorrhage in two eyes.

At surgery, ROP was Stage 4a in 29 eyes (74%), and Stage 4b in 10 eyes (26%).

The location of tractional retinal detachment (TRD) in Stage 4a ROP was in the temporal quadrant in seven eyes (24%), superior and inferior to the disc in six eyes (21%) each, and nasal to the disc in five eyes (17%). Radiating peripapillary folds were seen in five eyes (17%) due to prepapillary traction. In eyes with Stage 4b, the location of TRD was mainly over the posterior pole, with nasal dragging of macula in two eyes, and TRD extending to the temporal periphery in one eye. This localization of TRD did not affect the anatomical or functional outcome, but was helpful in deciding the plan of surgery, including the placement of sclerotomies.

Intraoperatively, adequate relief of traction was achieved in 37 eyes (95%). While significant intraoperative bleeding occurred in 13 (33%) eyes, only in two eyes, the bleeding was severe enough not to permit adequate dissection. Iatrogenic retinal breaks occurred in three eyes (8%), of which one developed total retinal detachment postoperatively, and underwent lensectomy and vitrectomy. The remaining two eyes underwent fluid-gas exchange, endolaser and perfluoropropane (C_3_F_8_) gas injection during the same sitting. All these three eyes attained favorable anatomical results at the last follow-up.

**Figure 1:**
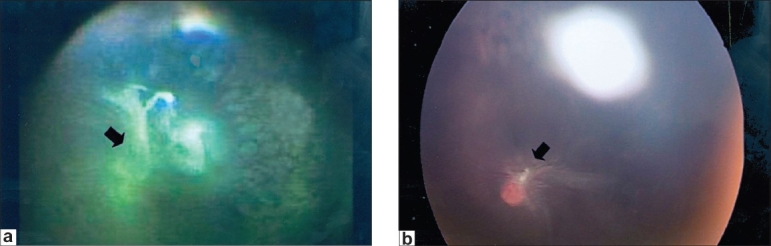
(a) Preoperative fundus photograph showing fibrovascular proliferation (FVP) and focal tractional detachment involving posterior pole (arrow). Note the well-ablated peripheral retina (b) Corresponding postoperative fundus photo of the same patient. Note the trimmed FVP at the disc with radiating dry retinal folds (arrow)

The mean follow-up period was 15 months (range 2–55 months). One patient (Stage 4a) was lost to follow-up after the surgery. At the follow-up examination, 28 of 38 eyes (74%) achieved a complete or posterior pole retinal reattachment with one procedure [Figures 1a and b]. These included 23 eyes (82%) with Stage 4a ROP and five eyes of Stage 4b ROP. Lens clarity was maintained in all eyes.

Of the 10 eyes (26%) with primary failures, seven underwent further surgery in the form of lensectomy and membrane dissection. In six of these, total or posterior pole reattachment was achieved postoperatively.

The mean birth weight and gestational age of babies with primary success was 1187.4 g and 28.5 weeks respectively, as compared to 1395 g and 30 weeks in primary failure cases. All the 10 patients with primary failures had at least two systemic diseases, and eight had at least three or more systemic diseases at birth. In general, the eyes which had a primary failure following surgery were found to have a stormier prenatal and natal period, with more aggressive and advanced disease at presentation.

The visual outcomes followed the anatomical trends. After one procedure, out of the 28 anatomically successful eyes, 24 had achieved good central, steady and maintained fixation. Out of these there were 20 eyes with Stage 4a and four with Stage 4b ROP. The final visual outcome was favorable in 30 of 38 eyes (79%). This constituted 24 of 28 Stage 4a ROP eyes (86%), and six of 10 Stage 4b ROP eyes.

Statistical analysis was performed using analytical software SPSS Version 14.0 (Chicago, Illinois). Variables like birth weight, gestational age, age at presentation and stage of ROP at surgery were studied using binary logistic regression analysis. Factors like sex, location of TRD, preoperative pre-retinal hemorrhage, preoperative vitreous hemorrhage, and intraoperative complications were studied using Chi square test. None of the factors studied were found to be statistically significant in terms of favorable anatomical or functional outcomes.

## Discussion

The surgical management of Stage 5 ROP has been unsatisfactory.[1,10] Stage 4 ROP was earlier managed with scleral buckling, however, this procedure had certain limitations. It did not restore normal retinal anatomy and was unsuitable for cases with very posterior disease, though it was satisfactory for relieving peripheral traction. Although the lens is spared during scleral buckling procedure, anisometropia can still occur and be a cause of amblyopia.[11,12]

Maguire *et al.*,[3] introduced the concept of lens-sparing vitrectomy which was more capable of relieving the posterior traction (Zone 1 and posterior Zone 2 ROP) and restoring near normal anatomy.[12]

In the present series, the initial few surgeries were performed with a two-port system, however, later we preferred a three-port system for lens-sparing vitrectomy in these eyes. It helped to maintain the intraocular pressure during the procedure and during the closure of other sclerotomies at the conclusion of surgery. Also, this system permits the surgeon to switch hands in order to perform anterior dissection without the risk of transient globe hypotony and lens touch. Previously, authors have shown a concern over the placement of the infusion cannula in the inferotemporal quadrant and rotating the eye into the same quadrant, which may cause lens injury either by a direct mechanical contact or by the hydrostatic forces from the infusion stream due to the small lid fissure.[6] However, we did not find this significant in our cases, as there was no lens injury and the lens remained clear in all the patients at the last follow-up visit, irrespective of whether two or three-port vitrectomy was performed.

This is one of the few studies providing the visual outcomes in addition to the anatomical outcomes and complications of LSV in Stage 4 ROP in the same paper. In the present series, the success has been good, with favorable anatomical outcome in 23 of the 28 eyes (82%) in Stage 4a and five out of 10 eyes in Stage 4b ROP with a single procedure. The visual results were favorable in eyes with successful retinal reattachment. The Stage 4a ROP eyes attained adequate vision in 71% (86% after second procedure) eyes. In Stage 4b, four out of 10 eyes had favorable functional outcome (six eyes after second procedure). It is evident that where the macula was involved in the detachment (Stage 4b), the results were less satisfactory, both anatomical and visual. Although the numbers were small to come to a definite conclusion, the difference in results points to the fact that anatomical results carry an implication on the functional results. The reported superior functional results after LSV for Stage 4a in the literature support our belief.[4,5,8]

Although the success rate with only Stage 4a is comparable, overall success rate in our series is lower compare to a few published reports.[4,6,8] Compared to other reported series, mean gestational age, birth weight and mean age at surgery was higher in our series. These factors might be responsible for the relatively lower success in our series. Even in a subgroup of primary failure in our series itself, the mean birth weight and gestational age was higher compared to the rest of the infants in the series. Yu *et al.*,[13] have also reported relatively lower success rate, 75.0% and 66.6%, in Stage 4a and 4b ROP respectively in their series with mean birth weight of 1224 g.

The pathoanatomy was found to be varied ranging from focal traction restricted to the ridge area to significant proliferation extending from the optic disc. In most cases there was vitreous schisis, with sheets of vitreous still adherent to the posterior retina while there were membranes mimicking posterior vitreous detachment adherent to the ridge. An attempt was made to peel all vitreous remnants from the retina, at least up to the ridge. The extent of intraoperative hemorrhage was also variable and not always predictable from the preoperative picture. Where fibrovascular tissue was trimmed, it could be cauterized but surface retinal ooze was left to stop on its own. Postoperatively some amount of vitreous hemorrhage was usually present but tended to clear in days to weeks. Re-surgery was done if the hemorrhage failed to clear in three to four weeks or if ultrasonography showed evidence of increasing TRD, instead of settling TRD.

In most cases the TRD settled rapidly and by six weeks postoperatively, the retina had near normal configuration barring the photocoagulation marks. Where residual traction was present, the configuration of the retina was to that extent distorted, depending on the location of the fibrous tissue [Figure 1b]. Surgical failure was due to re-proliferation leading to persistent or increasing TRD. In most of these cases, a lens sacrifice was needed to adequately remove the fibrosis in the second sitting.

## Conclusions

Lens-sparing vitrectomy has a decisive role in the management of eyes with ROP that have progressed to Stage 4 despite adequate laser photocoagulation. The results of LSV for Stage 4a and 4b ROP are very satisfactory in our series, both in terms of anatomical success and functional outcome, although this procedure is associated with a few intra- and postoperative complications.
